# HIV Reactivation from Latency after Treatment Interruption Occurs on Average Every 5-8 Days—Implications for HIV Remission

**DOI:** 10.1371/journal.ppat.1005000

**Published:** 2015-07-02

**Authors:** Mykola Pinkevych, Deborah Cromer, Martin Tolstrup, Andrew J. Grimm, David A. Cooper, Sharon R. Lewin, Ole S. Søgaard, Thomas A. Rasmussen, Stephen J. Kent, Anthony D. Kelleher, Miles P. Davenport

**Affiliations:** 1 Centre for Vascular Research, University of New South Wales, Sydney, Australia; 2 Department of Infectious Diseases, Aarhus University Hospital, Aarhus, Denmark; 3 Kirby Institute, University of New South Wales, Sydney, Australia; 4 Peter Doherty Institute for Infection and Immunity, Melbourne, Australia; 5 Department of Infectious Diseases, Alfred Hospital and Monash University, Melbourne, Australia; 6 Department of Microbiology and Immunology, The University of Melbourne at the Peter Doherty Institute for Infection and Immunity, Melbourne, Australia; University of North Carolina at Chapel Hill, UNITED STATES

## Abstract

HIV infection can be effectively controlled by anti-retroviral therapy (ART) in most patients. However therapy must be continued for life, because interruption of ART leads to rapid recrudescence of infection from long-lived latently infected cells. A number of approaches are currently being developed to ‘purge’ the reservoir of latently infected cells in order to either eliminate infection completely, or significantly delay the time to viral recrudescence after therapy interruption. A fundamental question in HIV research is how frequently the virus reactivates from latency, and thus how much the reservoir might need to be reduced to produce a prolonged antiretroviral-free HIV remission. Here we provide the first direct estimates of the frequency of viral recrudescence after ART interruption, combining data from four independent cohorts of patients undergoing treatment interruption, comprising 100 patients in total. We estimate that viral replication is initiated on average once every ≈6 days (range 5.1- 7.6 days). This rate is around 24 times lower than previous thought, and is very similar across the cohorts. In addition, we analyse data on the ratios of different ‘reactivation founder’ viruses in a separate cohort of patients undergoing ART-interruption, and estimate the frequency of successful reactivation to be once every 3.6 days. This suggests that a reduction in the reservoir size of around 50-70-fold would be required to increase the average time-to-recrudescence to about one year, and thus achieve at least a short period of anti-retroviral free HIV remission. Our analyses suggests that time-to-recrudescence studies will need to be large in order to detect modest changes in the reservoir, and that macaque models of SIV latency may have much higher frequencies of viral recrudescence after ART interruption than seen in human HIV infection. Understanding the mean frequency of recrudescence from latency is an important first step in approaches to prolong antiretroviral-free viral remission in HIV.

## Author Summary

During treatment of HIV infection the virus persists in infected cells in a quiescent or ‘latent’ state. If treatment is stopped, then virus rebounds to detectable levels usually within 2–3 weeks. This is thought to occur due to release of infectious virus from a reservoir of long-lived latently infected cells. Reducing the number of latently infected cells should allow a prolonged period of HIV remission without antiviral treatment. A fundamental question is ‘how frequently does infectious virus emerge from the pool of latently infected cells?’, and thus how much would we need to reduce the number of latently infected cells to produce remission? Here we directly estimate the frequency of successful viral reactivation in four independent cohorts of patients undergoing treatment interruption. We find that active infection is initiated on average once every 5–8 days, considerably more slowly than previously thought. This has important implications for how much we need to reduce the number of latent cells in order to produce remission. Whereas previous analyses suggested that we would need to reduce the latent cell number 2000 fold to produce an average one-year remission, we show that reducing the latent cell number by 50–70 fold could achieve this aim.

## Introduction

The development of highly potent antiretroviral therapy (ART) for HIV means that the virus can be effectively controlled in most treated patients. However, ART must be taken continuously, as interruption of ART is followed by the rapid recrudescence of virus from a quiescent ‘latent reservoir’ of infected cells. A major thrust of HIV research is to reduce the latent reservoir so that prolonged antiretroviral-free HIV remission can be achieved. A number of ‘latency reversing agents’ (LRA) are currently being developed to reduce the latent reservoir by reactivating latently infected cells [1–4]. Clinical studies of LRA in HIV-infected patients on ART have shown the ability to significantly increase cell-associated unspliced HIV RNA and in some studies increase plasma HIV RNA. However, these studies have not resulted in decreases in HIV DNA—a crude surrogate marker of latently infected cells—or measurable reductions in various measurements of the latent reservoir or antiretroviral free HIV remission [5–10]. A fundamental question in achieving HIV remission is what level of reduction of latently infected cells is required? It is currently estimated that the reservoir of latently infected cells may be between one and 60 million cells [11–13]. Complete elimination of this would thus require reducing the size of the reservoir by at least one million fold. However, reducing the reservoir by smaller amounts may still produce significant delays between ART-interruption and viral recrudescence, allowing potentially for prolonged interruptions of therapy before viral recrudescence. Understanding factors that predict the duration of viral remission will be critical for the future design of eradication studies [14].

The dynamics of HIV reactivation from latency have not been examined in detail experimentally. However, it is clear from a number of studies that after ART-interruption there is generally a delay of about a week before viral rebound can be detected, and about half of the patients often experience rebound within the first two weeks or so [8,15–17]. However, a proportion of patients usually remains virus-free even after a month, suggesting variable dynamics of reactivation. From this observation, we can attempt to predict the underlying dynamics from our understanding of infection. Firstly, we expect that the latently infected cells might ‘attempt’ reactivation even during successful ART, because ART itself is not expected to affect the rate of initial latent cell reactivation. These reactivation attempts by latent cells may occur at some average frequency, and some fraction of these events will produce replication competent virus and thus be capable of initiating successful viral rebound. These reactivation events do not result in successful viral growth while therapeutic levels of ART are present. Thus, after ART there will be a period of ‘drug-washout’ before the virus is able to grow (which will vary depending on the pharmacokinetics of the ART regime). Once ART levels have declined sufficiently that viral growth is possible, there may be some delay until the first replication-competent viral reactivation event occurs (assuming reactivation is occurring randomly). Following the first successful reactivation, we expect virus levels will start at some low level, and then take some time to grow to the level of viral detection. Together, the time for drug-washout and viral growth create a ‘fixed delay’ before reactivation can be detected, and likely explain the fact that little rebound is usually detected in the first week after ART-interruption. After this fixed delay, if latent cells are reactivating randomly at some average frequency, this will lead to an exponential distribution in the time-to-reactivation observed, and may explain why some patients remain virus-free for longer periods. By fitting of the time-to-reactivation curve, we can estimate the frequency of successful reactivation from latency.

In this study, we directly estimate the frequency of HIV recrudescence from latency following ART-interruption by analysing the time to detection of viral rebound from 4 independent patient cohorts undergoing ART-interruption. We find that the average frequency of successful reactivation from latency is approximately once every 6 days, around 24 times lower than previously estimated [18,19]. This low rate of successful reactivation has important implications for designing future eradication studies.

## Results

### Frequency of HIV reactivation after ART interruption

**Fig 1:**
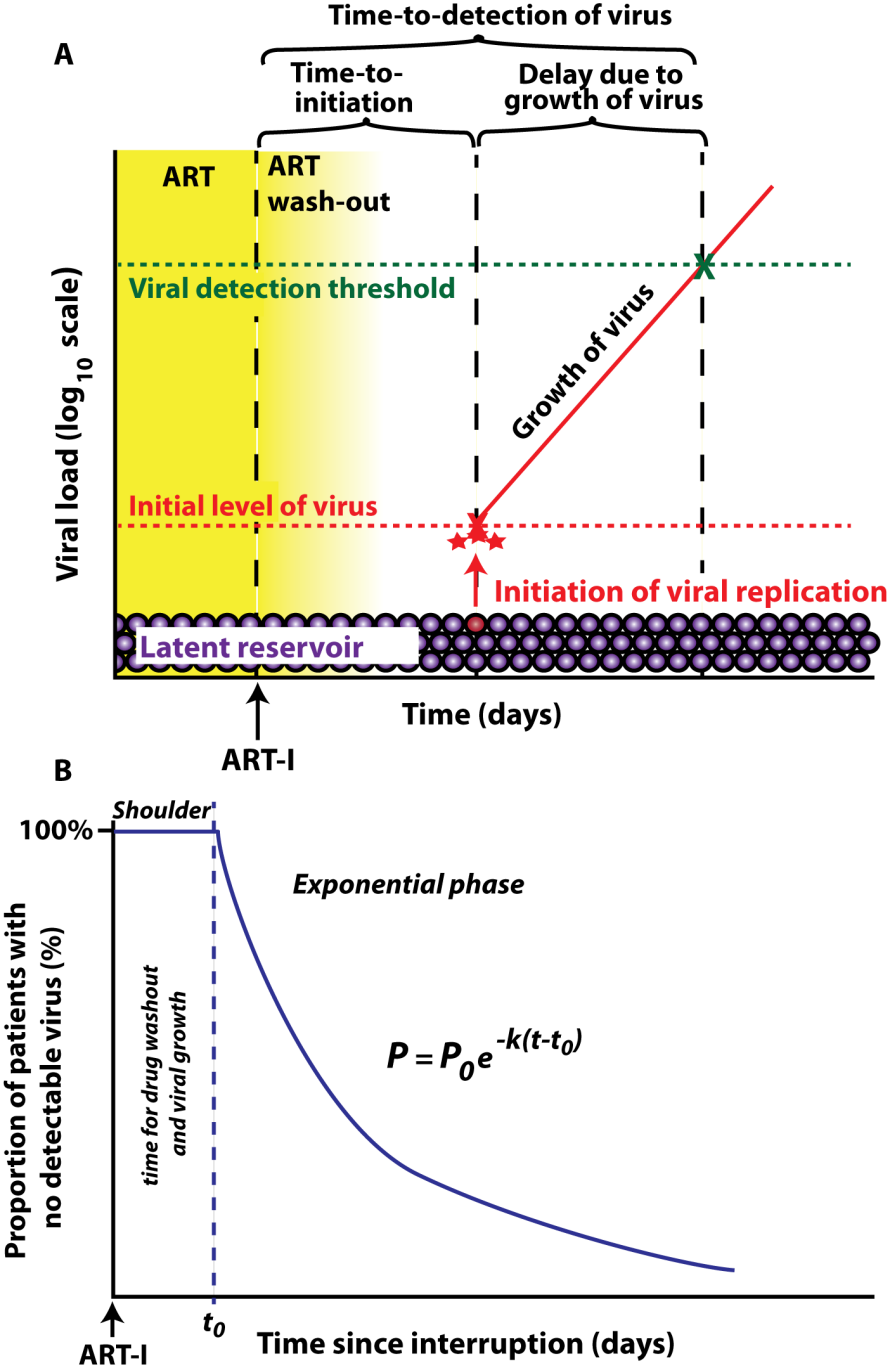
Schematic of viral recrudescence after ART-interruption. (A) After ART-interruption, there is an initial delay when virus cannot grow, due to ‘washout’ of ART. Once viral replication is possible, there is a variable time until successful viral replication is initiated from the latent reservoir. The low initial level of replicating virus then increases until it reaches the detection threshold. Time to initiation refers to time until replicative infection commences, which is followed by a delay due to viral growth, and then by detection of plasma virus. (B) time-to-detection plotted as a ‘survival curve’. The initial shoulder occurs due to ART washout and viral growth, and is followed by an exponential decay in the proportion of patients with no detectable virus.

After interruption of successful ART, HIV rebounds to detectable levels within a few weeks in the majority of patients. This requires reactivation of latent cells bearing replication competent virus. The frequency with which this reactivation occurs is likely a function of the size of the latent reservoir (which may vary substantially between individuals [20–22]), and the per-latent-cell probability of successive reactivation. If the initiation of viral growth after ART-interruption is a random event then the distribution of time-to-initiation will be exponential, and we could estimate the average frequency of initiation directly from the ‘survival curve’ of time-to-initiation of viral growth. However, since we are usually unable to detect the initiation of viral growth after ART-interruption, we instead measure ‘time-to-detection of virus’ at some threshold viral level. The actual time when we first detect virus is delayed both because of drug washout preventing viral growth immediately after interruption, and the time taken for the virus to grow from its level at initial reactivation to our threshold for detection of plasma virus (Fig 1A). The duration of the delays due to drug washout and viral growth to the level of detection only affect the “shoulder” of the curve of time-to-detection by delaying the time until we could first detect virus (Fig 1B). The average delay to viral detection is thus the sum of the average time between initiation of successful reactivation events, and the delay from reactivation to detection. There may be a distribution in both time-to-initiation as well as from reactivation to detection, both of which may affect the shape of the subsequent time-to-detection curve.

**Fig 2:**
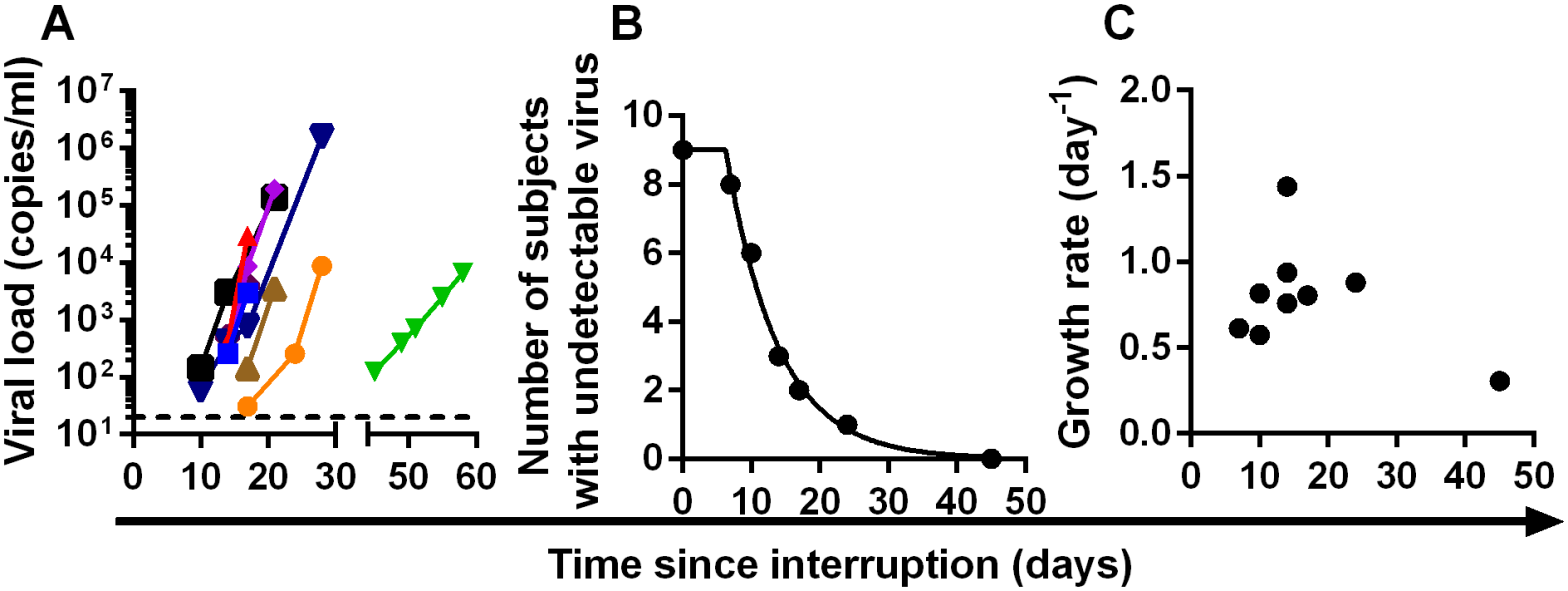
Dynamics on recrudescence in nine patients enrolled in Panobinostat trial (reference [8]). (A) The trajectory of viral load of individual patients after ART-interruption. Dashed line indicates threshold of detection (20 copies ml^-1^) (B) the ‘survival curve’ of patients without detectable virus. The frequency of recrudescence is equivalent to one initiation of viral replication occurring every 7.6 days. (C) growth rate of virus after detection is not significantly correlated with time to recrudescence (p = 0.9).

To test this approach we first analysed the kinetics of time-to-detection of HIV in a published cohort of nine patients treated with the LRA panobinostat, and undergoing therapy interruption and biweekly monitoring of viral loads [8]. The threshold of detection of HIV viremia was 20 copies ml^-1^, and virus was first detected between day 10 and day 45 across the patient group (Fig 2A). To see if the observed time-to-detection was consistent with an exponential process, we plotted the ‘survival curve’ of time-to-detection in the cohort (Fig 2B). This plot demonstrates an initial shoulder (as expected due to drug washout and the time taken for viral growth), followed by a survival curve that conformed well to an exponential process. The exponential rate can be estimated from the survival curve, and equates to a frequency of viral reactivation of once every 7.6 days (95% confidence intervals (CI) = 6.5, 9.1).

To test whether the exponential model was suitable, we performed a Chi-squared goodness-of-fit analysis, which indicated a good fit to the data (p = 0.988). Although the analysis above is consistent with an exponential process, this does not prove that this is the only source of delay. It has also been proposed that early stochastic events, differences in the initial level of replicating virus, or differences in viral growth rate may contribute to the delay until viral detection [18,23]. However, a comparison between a survival curve based on an exponential distribution with one based on a gamma distribution showed that the gamma distribution (which incorporates multiple delays) did not provide a significantly better fit (p = 0.72 F-test). We can also use a modelling approach to understand the effects of these different factors on time-to-detection. For example, Pearson *et al*. [24] have estimated the distribution of delays arising from early stochastic events following primary HIV infection under different assumptions. Under most scenarios, the expected distribution of delays from early stochastic events is of the order of 1–3 days. Moreover, the importance of stochastic delays only becomes relevant in the presence of a low frequency of reactivation. In the presence of frequent reactivation, the stochastic delay in any individual reactivation event is overcome by the rapid arrival of the next reactivation (see S1 File). Another potential cause of differences in time-to-detection is differences in initial levels of virus. That is, if the first latent cell to reactivate were to ‘seed’ the infection with a lower initial level of virus, then it will take longer for the virus to grow to the level of detection. However, given the growth rates of virus observed in these patients, the initial level of virus would have to vary by many orders of magnitude to produce the delays observed (see S2 File).

It is also possible that the distribution in time-to-detection arose because of slower viral growth in some patients. To investigate whether the observed differences in detection times could be due to slower viral growth, we estimated the viral growth rate from the serial viral load measurements after virus became detectable, and investigated whether later detection was associated with slower viral growth. We found no correlation between viral growth rate and when virus was first detected (Fig 2C), indicating that slow viral growth did not explain the distribution of time-to-detection.

Taken together, these results are consistent with the observed time-to-detection in this cohort being determined by a low rate of viral recrudescence from latency, with an average frequency of initiating viral replication of once every 7.6 days. A potentially confounding factor with this analysis is that patients were part of a trial of the LRA panobinostat, a histone deacetylase inhibitor, to assess its effect on the HIV reservoir under ART. We note that although panobinostat increased HIV in plasma and cell-associated unspliced HIV RNA, there were no changes in HIV DNA. Thus, it seems unlikely that panobinostat treatment significantly reduced the HIV reservoir. Alternatively, it is possible that panobinostat-induced activation might increase the frequency of viral recrudescence, although this seems unlikely given that the last dose of panobinostat was administered >36 weeks before ART interruption.

### HIV reactivation in other cohorts

**Fig 3:**
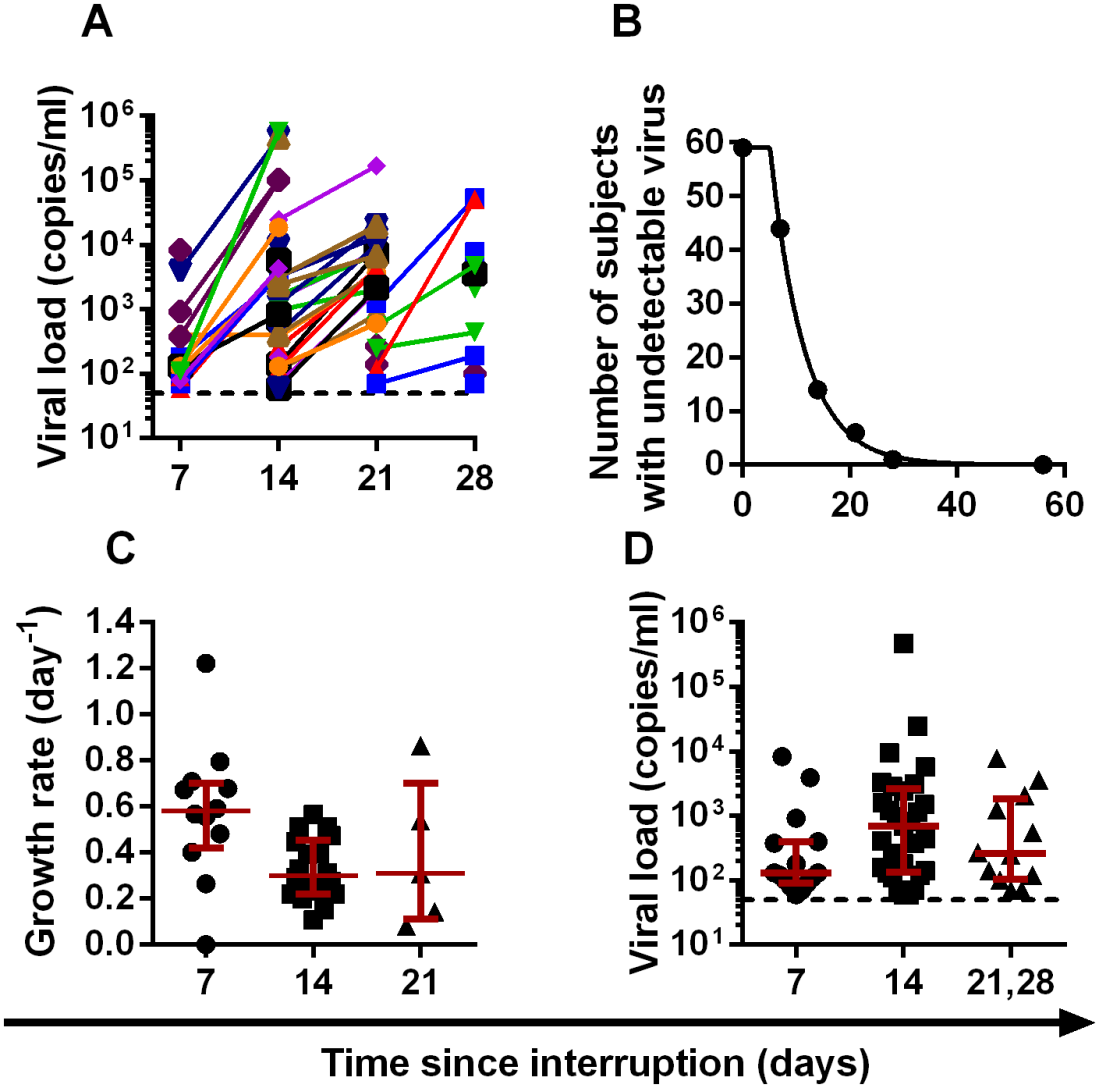
Dynamics of recrudescence in 59 patients enrolled in the Pulse study (reference [15]). (A) Trajectory of viral load in individual patients. Dashed line indicates threshold of detection (50 copies ml^-1^) (B) survival curve of time to detection (frequency of initiation of viral replication is once every 6.3 days). (C) Growth rate estimated from first two weeks of detectable virus. (D) Average viral load at detection for patients detected at different weeks. Dashed line indicates threshold of detection (50 copies ml^-1^)

Given the small number of patients in cohort 1 and their prior treatment with an LRA, it is important to confirm the estimated frequency of reactivation in other patient cohorts that have not received LRA. Therefore we obtained data on time to recrudescence for another three cohorts of patients undergoing ART-interruption, comprising an additional 91 subjects (summarised in Table 1). In the second cohort, 59 patients treated in primary infection underwent treatment interruption and weekly monitoring [15] (Fig 3A). Estimating the frequency of initiation from the time-to-detection of virus (at a threshold of 50 copies ml^-1^) we found an average frequency of once every 6.3 days (CI = 5.7, 7.1) (Fig 3B), similar to our estimate from the panobinostat cohort. Estimation of viral growth rate was less accurate in this cohort, as patients were only sampled weekly. We compared viral growth rate in this cohort with the time-to-detection to once again check whether a difference in viral growth rate could explain the different time-to-detection of virus. We estimated viral growth using a ‘two-point’ growth estimate to compare growth rates of virus in patients where virus was first detected in different weeks. Using this approach, there was no difference in growth rate estimates for patients with virus detected in weeks two and three, but a slightly higher growth rate in week one (Fig 3C). However, this estimate of growth rate is biased by the fact that viral loads at detection were lower in week one, and therefore we were estimating viral growth rate earlier in the growth phase, before it slows towards peak (Fig 3D). Overall, differences in viral growth did not appear to play a major role in time-to-detection of infection in this cohort.

**Table 1:** Summary of cohorts.

Tables: Anti-Retroviral Treatment Interruption (ART-I) trial	Subjects	Treatment	Viral detection	Frequency of reactivation.
Cohort 1: Panobinostat trial [8]. 9 patients with ART-I	Chronic HIV infection*	Suppressive ART** for 30–147 months with <20 HIV RNA copies ml-1 Panobinostat 20 mg three times per week, every other week for 8 weeks. Interruption at >36 weeks from last Panobinostat.	Twice weekly sampling with 20 copies ml-1 detection threshold.	Every 7.6 days.
Cohort 2: PULSE trial [15] 59 patients with ART-I.	Primary HIV infection (<4 bands on Western blot, or positive Western blot + negative HIV test in last 6m)	Suppressive ART*** for 6–12 months treatment with <50 HIV copies ml-1. Randomised 1:1 to receive hydroxyurea (500mg daily).	Weekly sampling for first month with 50 copies ml-1 detection threshold.	Every 6.3 days.
Cohort 3: (reference [16]) ART + / − earlier IL-2 administration. 18 patients with ART-I.	Chronic HIV Infection	Suppressive ART**** for >59 weeks with VL <500 copies ml-1. 12 Patients received IL-2 therapy.	50 copies ml-1 threshold with bDNA assay. Time to 50 copies estimated by extrapolation.	Every 5.1 days.
Cohort 4: Swiss-Spanish Intermittent Treatment Trial (reference [17]). 14 patients with five ART-I.	Chronic HIV Infection	Suppressive ART***** for 11–32 months treatment with <50 HIV copies ml-1.	Sampling day 4, 8, 14, with 50 copies ml-1 detection threshold.	Every 7.2 days.
SIV infected macaques [26]	SIVmac251 infected rhesus macaques treated at days 7, 10, and 14 post-infection (n = 4 for each)	Tenofovir, emtricitabine, dolutegravir for 24 weeks	Sampling twice weekly, with 50 copies ml-1 detection threshold.	Every 1.7 days

* One subject was treated with ART early after diagnosis of unclear duration of HIV infection, the other 8 subjects were infected for 390–6574 days from diagnosis until ART begun.

** Tenofovir, Emtricitibine and either Rilpiverine (n = 3) or Efavirenz (n = 4) or Raltegravir (n = 1). One subject received Zidovudine, Lamivudine and Abacavir.

*** Indinavir, ritonavir, didanosine and either stavudine or lamivudine.

**** zidovudine, lamivudine plus either indinavir (n = 7) or nelfinavir (n = 1) or ritonavir (n = 1); stavudine, lamivudine plus nelfinavir (n = 2); stavudine, didanosineplus nelfinavir (n = 2); zidovudine, didanosine plus nelfinavir (n = 1).

*****including 3, 4, and 5 drug regimes.

**Fig 4:**
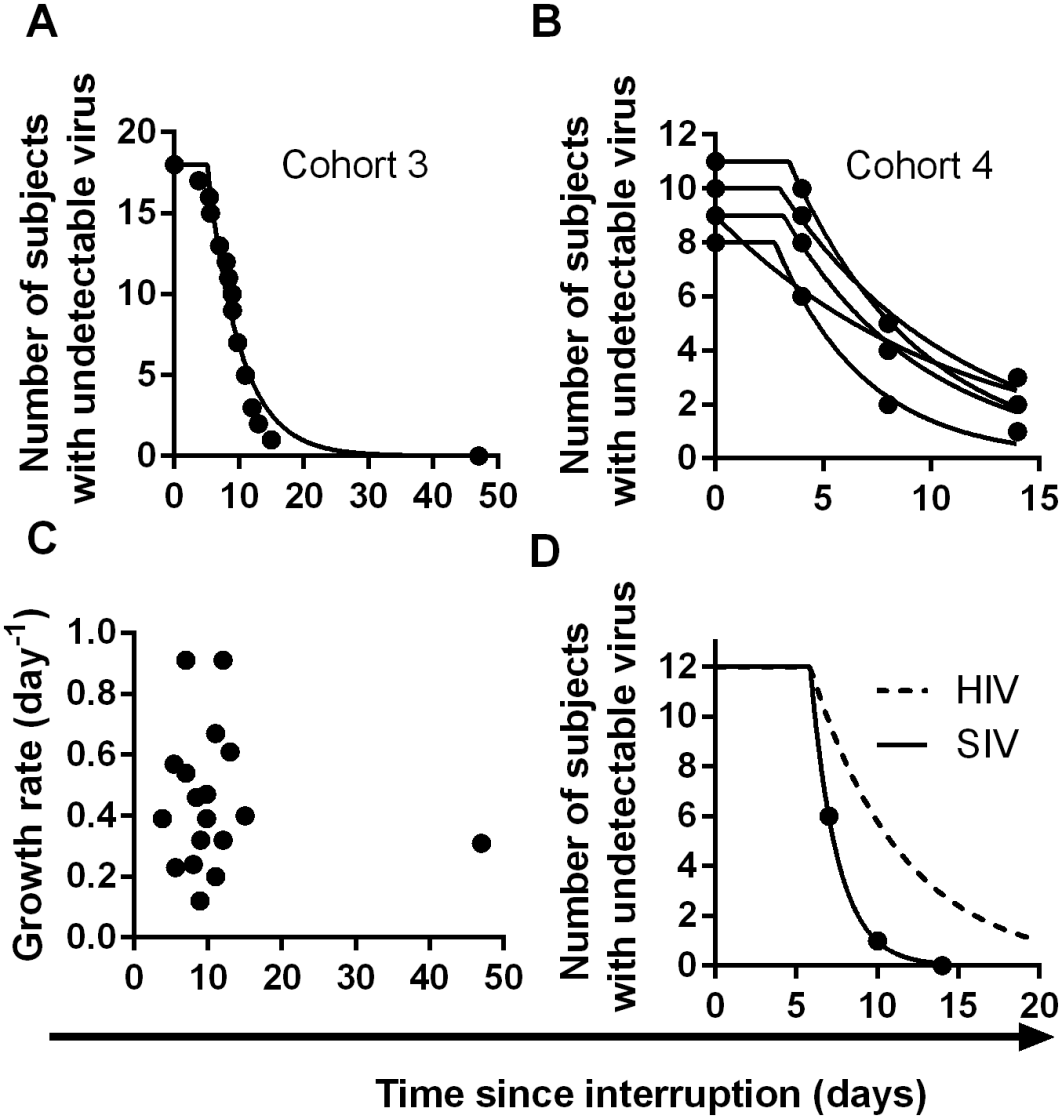
Time-to-detection of virus in cohorts 3 and 4. (A) time-to-detection in cohort 3, of 18 patients undergoing interruption (reference [16]). The best-fit frequency of reactivation is once every 5.1 days. (B) time-to-detection in cohort 4, of 14 patients undergoing five interruptions, and monitored at days 4, 8, and 14 (reference [17]). The best-fit frequency over all interruptions is once every 7.2 days. (C) Time to recrudescence is not correlated with growth rate in cohort 3. (D) Higher reactivation rates in SIV than HIV. The estimated frequency of initiation of viral replication in SIV infected macaques treated with ART between 7 and 14 days post-infection (from reference [26]) is shown as solid line, and was found to be once every 1.7 days. The best-fit frequency of reactivation across the four HIV cohorts (a reactivation event every 6 days) is shown as a dashed line.

We also analysed two other cohorts using data extracted from earlier publications on ART-interruption. The third cohort included 18 patients undergoing ART-interruption, where time-to-detection at a threshold of detection of 50 copies ml^-1^ was measured and viral growth rates were estimated (Table 2 of reference [16]). The fourth cohort included 14 patients monitored on days 4, 8 and 14 following ART-interruption (using data from Fig 1 of reference [17]). Because of the small number of patients and timepoints in the fourth cohort, we included data from five sequential interruption cycles. Using the same method for estimating the frequency of initiation from the time-to-detection curves, we found very similar frequencies of viral recrudescence in these two cohorts (every 5.1, days CI (4.2, 6.5) and 7.2 days CI (6.0, 8.7) respectively, see Fig 4A–4B). In the third cohort viral growth rate was also estimated independently in the original study (reference [16], Table 2), and again, viral growth rate was not significantly correlated with time-to-detection of infection (Fig 4C), confirming that differences in viral growth played little role in the time-to-recrudescence in this study. Comparing all cohorts together, we found a trend for slightly higher frequencies of reactivation in cohort 3, who initiated ART in chronic infection, and slightly lower frequencies of reactivation in patients treated in primary infection (cohort 2) or with the LRA panobinostat (cohort 1). However, the frequency of recrudescence was not significantly different between the cohorts (*p*- value = 0.059, F-test). In addition, we used a Chi-squared test to assess whether the exponential model of reactivation frequency was suitable across the four datasets, and found that the data conformed well to this model (p = 0.996) and that a survival curve based on a gamma distribution did not provide a better fit (p = 0.5, F-test).

Overall, despite the different sampling regimens and study designs, the estimated frequencies of reactivation were similar across the four cohorts studied (once every 5.1, 6.3, 7.2, and 7.6 days), with an average frequency of once every 6.0 days (CI 5.5,6.6).

### Ratios of ‘reactivation founder’ virus following ART-interruption

The analysis of time-to-detection of HIV following ART-interruption suggests a relatively low frequency of recrudescence from latency, and thus a significant delay between successive reactivation events. If each reactivation event is ‘founded’ by virus produced by a single latently infected cell, this predicts that early after ART-interruption, the viral population would often be the progeny of a single latent cell (in much the same way as virus observed early after sexual transmission is thought to arise from a single founder virion). Joos *et al* [25] have compared the diversity of the HIV plasma viral population present soon after ART interruption with the diversity present prior to commencing ART. They found a major narrowing of diversity after ART-interruption, suggesting monoclonal or oligoclonal origins of the plasma virus. Although the viral population after ART-interruption was not entirely homogeneous, they observed one or more ‘families’ of closely relate viruses, differing by only a few nucleotides, similar to the founder viruses observed after sexual transmission. They concluded from this that the viral population after ART-interruption represented random reactivation of latently infected cells, rather than continual seeding of virus.

We accessed the viral sequence data from the Joos study (Genbank accession numbers listed in the original publication [25]) and reanalyzed this data in order to investigate the ratios of different ‘reactivation founder’ viruses in these patients. We observed six patients in whom it was possible to identify and count the frequency of founder viruses early after ART-interruption, and investigated the ratio of the number of copies of the most frequently observed founder to the next most frequently observed founder (see S3 File). This ratio of founder copies is determined by both the delay until the next founder starts growing, and the overall growth rate of the virus. We then considered the distribution of these founder ratios, and used this to estimate the distribution of reactivation events and thus the average frequency of reactivation.

**Fig 5:**
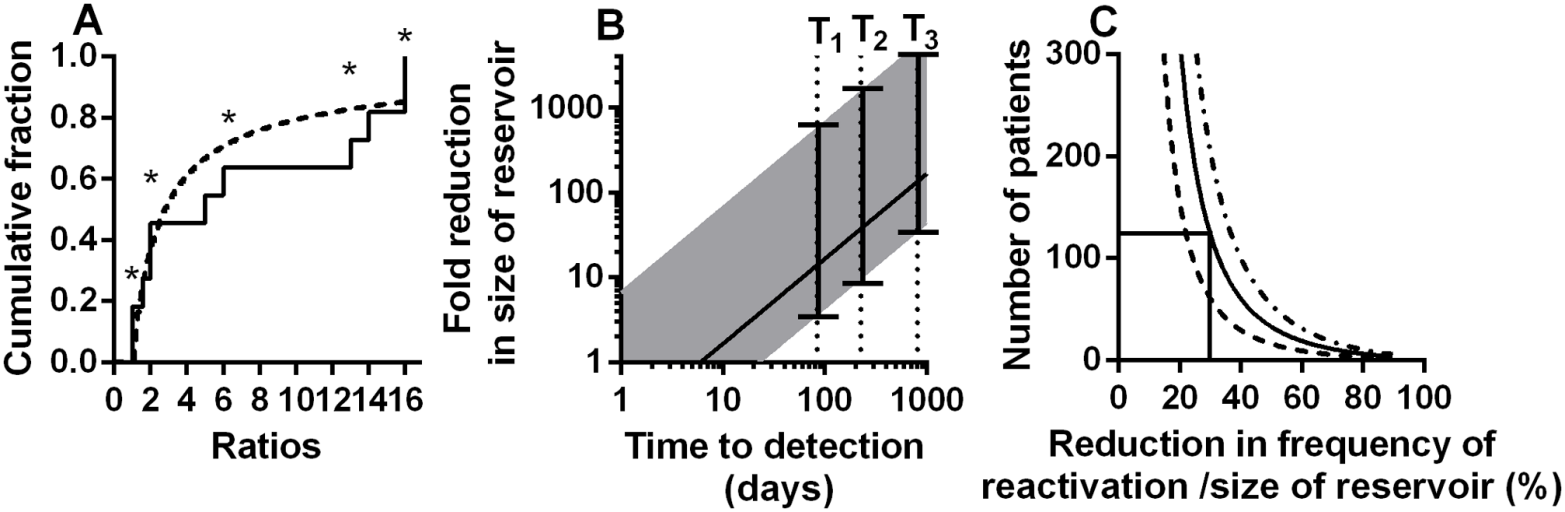
Modelling of kinetics of time-to-detection: A: Using the ratio of the number of copies of reactivation founder viruses to estimate rate of initiation of viral growth. The cumulative proportion of founders with different ratios (to the size of next largest founder) is shown. Solid line is ratios from the experimental data, dashed line is the theoretical distribution with the best fit frequency of rebound of once every 3.6 (CI 1.98–6.62) days. Ratios marked with an asterisk are where we could only estimate a minimum ratio (ie: there was no detected next founder virus). (B) Estimating the reduction in frequency of recrudescence (and reservoir size) from observed delay to detection of virus. For a ‘normal’ reservoir size, we find an average frequency of reactivation of once every 6 days (which also equates to an average delay to reactivation of 6 days). For patients with longer time-to-detection we can estimate the relative size of the reservoir compared to our cohort populations. Solid line shows the fold reduction in reservoir size that would be estimated simply comparing the observed time to detection with the estimated average of 6 days. Since reactivation does not always occur at the average time, the range expected for 95% of subjects is shown (shaded area). T_1_ and T_2_ are the delays for two patients that underwent allogeneic stem cell transplantation (reference [30]). T_3_ is the delay observed in the ‘Mississippi baby’ (reference [29]). (C) Difficulties using treatment interruption studies to measure changes in the reservoir. The number of patients required (y-axis) to have a 50% (dashed line), an 80% (solid line) or a 95% (dot-dash line) power to detect a given reduction in reservoir (x-axis) is shown, based on Log-rank test. This assumes a 100-day follow up after ART-interruption.

We used maximum likelihood estimation to fit the ratios of founder copies observed in the Joos study to the theoretical distribution of ratios we would expect if founders reactivated λ times per day (described in detail in methods). We found the average frequency of reactivation events (1/λ) to be once every 3.6 days (CI 1.98–6.62 days). The real delay between reactivation events is likely more than this, because in some cases (marked with an asterisk in Fig 5A) we can only estimate the minimum ratio (for example, if all 16 sequences in a patient are from the same founder we can only say the frequency of the next founder is likely <1/16, whereas it could be much lower). On the other hand, it is also possible that two latent cells bearing founder viruses that were identical in the sequenced region reactivated sequentially, and thus were classified as a single founder. We aimed to minimise the likelihood of this occurring by only selecting patients for this analysis with sufficient diversity of virus pre-treatment (sequenced in the same region of the virus). In addition, it is likely that the latent reservoir was indeed more diverse than the circulating virus immediately before treatment, as it contains an archive of different viral strains. Thus, it seems unlikely that we are aggregating multiple identical founder viruses. Future studies using larger regions of the virus, and / or more in-depth sequencing approaches should provide more accurate estimates of the ratio of reactivation founders and the frequency of reactivation. However, our analysis of the ratios of reactivation founder viruses leads to very similar estimates of reactivation frequency to those obtained studying time-to-detection.

### Frequency of SIV recrudescence in macaques

Recent studies in macaques have suggested that very early treatment after SIV infection may also lead to delayed time-to-recrudescence after ART-interruption [26]. In this study they found that time-to-recrudescence was very short and significantly correlated with area-under-the-curve of viral load since infection, although significantly longer delays were seen only animals treated within 3 days of infection [26]. Using the same approach to estimate frequency of reactivation from time-to-detection of virus in the animals treated with ART at days 7, 10 and 14 (ie: excluding animals treated at day 3), we found that the average frequency of initiation of viral replication in macaques was once every 1.7 days, compared to every 6 days in HIV (Fig 4D). One explanation for this might be higher levels of HIV DNA in the macaques. However, the total number of HIV copies per million PBMC measured in the macaques just prior to ART-interruption seems similar to that reported in patients during ART [8,27]. Another reason for the higher reactivation rate in macaques may be the generally shorter periods of treatment (24 weeks of ART in SIV versus >12 months in the HIV studies (Table 1)), which may have allowed less time for activated cells to decay and a steady-state of latently infected cells to be attained [28]. Alternatively, differences in immune activation or cytokine levels may also play a role. Regardless of the mechanism, this work suggests that short-term treated macaques may experience much higher rates of reactivation from latency compared to HIV patients even if treated early after infection.

### Modelling antiretroviral-free HIV remission

The primary goal in tackling HIV latency is to allow prolonged HIV remission in the absence of ART. Thus, a major question is how much we would need to decrease the latent reservoir in order to produce a durable delay in time-to-recrudescence and subsequent recommencement of ART? A previous study estimated that a reduction in reservoir size of >2000 fold would be required to provide a one year average delay until reactivation [18]. However, that study assumed that viral reactivation was over 24 times faster than our estimates (reactivation every 0.25 days), based on indirect modelling approaches published previously [18,19]. Our analysis demonstrates a much lower rate of viral reactivation, and thus much smaller reductions in the size of the latent pool would be needed for a one-year delay to reactivation. The required reduction in latent reservoir can be calculated as:

$$R=\frac{T}{d}$$ (1)

Where *R* is the required reduction in size of the latent reservoir, *T* is the length of delay until viral recrudescence and *d* is the average time between viral reactivation events (ie: the baseline frequency of viral reactivation). For a baseline frequency of reactivation of once every 6 days (the average over the four cohorts), our analysis predicts that a 61-fold reduction in the reservoir would provide an average one-year delay until recrudescence. Thus, for example, 12 rounds of therapy using an LRA that reduced the reservoir (and reactivation rate) by 30% would achieve an ≈72-fold reduction in the reservoir and hence an average one year ART-free control of viremia.

### Case studies of delayed viral detection

Several recent case reports have suggested that very prolonged remission is possible if the reservoir can be reduced by early treatment or other interventions such as bone marrow transplantation. In the case of the ‘Mississippi baby’, viral recrudescence was not observed until 27 months after ART-interruption [29]. Similarly, in two cases of haematopoietic stem cell transplantation in adults, viral recrudescence was not observed until 84 and 225 days after ART-interruption [30]. Our analysis indicates that the average frequency of reactivation is once every 6 days across the four cohorts we analysed. Therefore these three cases are respectively 135, 14 and 37 fold longer than expected on average in these cohorts. One might speculate from these delays that the reactivation rate and reservoir size were respectively 135, 14 and 37 fold smaller than average (using Eq 1). However, since reactivation is a random process, recrudescence is not always observed at the average time expected. Using the data we can estimate bounds for the likely frequency of initiation of viral replication (and the extent of reservoir reduction) based on observed time-to-detection of virus (see Fig 5B). For example, given an observed time-to-detection of 84 days or more, it is highly unlikely that the reservoir was of the average size determined by analysis of our four cohorts (probability for this is 8.4x10^-7^). For a time-to-detection of 84 days to lie within the range expected for 95% of subjects, then the average frequency of recrudescence would have to be bounded below by 23 days and above by 3318 days. This suggests that in this case the latent reservoir was most likely between 3.8 fold and 553 fold smaller than the average size estimated from our four cohorts. Using the same approach in the case of the Mississippi baby, the maximum predicted reduction in viral reservoir (top border of 95% CI) is 5,300 fold. A corollary to these observations is that the rate of reactivation from latency and level of viral reservoir in the transplant patients is not decreased as much as might be predicted from the degree of chimerism seen in peripheral blood (<0.001% of PBMC were of donor origin [30]). However, as noted by the authors of that study, the degree of chimerism in the patients’ tissues are likely significantly higher than that seen in PBMC [30] particularly as the patients received a reduced intensity conditioning regime. In addition, a number of recent studies have suggested that lymphatic sites may be a significant source of virus under therapy [31–33]. Thus, we speculate that reactivation from chimeric tissue sites might contribute to the observed reactivation rate. Overall, the wide error bars on estimates of potential reservoir size based on time-to-detection of individual patients (Fig 5B) suggest significant limitations in the use of time-to-detection to estimate reservoir size. Therefore, we also investigated the usefulness of time-to-detection assays in detecting the effects of LRA.

### Use of treatment interruption studies to measure reservoir purging

A major question in clinical trials of LRA is how to measure changes in the latent reservoir. Approaches using detection of plasma HIV RNA, cell-associated HIV RNA, cell associated HIV DNA, as well as *ex vivo* quantitative outgrowth assays have been studied [7–10,34,35]. However, it is not clear whether these measures will reflect time to viral recrudescence after ART-interruption *in vivo* [8,36]. Since HIV remission involves essentially a prolonged time-to-detection of virus, direct measurement of time-to-detection following ART-interruption will ultimately be the most clinically meaningful endpoint.

Treatment interruption studies to measure time-to-detection pose a number of ethical questions. Firstly, frequent treatment interruptions may increase morbidity or mortality compared to continuous treatment [37], although it is less clear that occasional interruptions would have the same effect. Secondly, interruption may act to ‘replenish’ the viral reservoir, although this does not appear to occur quickly [22]. Thirdly, such studies would ideally require a control group, in order to compare time-to-detection in treated versus untreated patients. However, in addition to these factors, there are also a number of issues with the statistical power to detect delays in time-to-detection. Firstly, as indicated by the extremely wide error bars in our estimates of relative reservoir size in the Boston patients and Mississippi baby (Fig 5B), time-to reactivation is not a useful measure of reservoir size in an individual, because of the random nature of reactivation. Time-to-detection is only useful in cohorts of patients. Once we understand time-to-detection as an exponential process, we can apply a power analysis to estimate how many patients would be required to identify differences in time-to-detection. Such an analysis suggests that to detect a 30% decrease in the reservoir (and a 30% increase in the frequency of initiation of viral replication) assuming a 100 day follow-up one would require >120 patients in each arm to have an 80% chance of detecting a difference (Fig 5C). Thus, such studies are only likely to be useful in detecting rather large changes in the reservoir and rate of reactivation.

## Discussion

Our work provides the first direct estimates of the frequency of viral recrudescence from latency based on analysis of time-to-detection of plasma viremia from ART-interruption cohort studies. Previous studies have modeled the recrudescence of virus following ART-interruption, using a variety of approaches. This work has often focused on the dynamics of virus within the individual, and either did not estimate the frequency of viral reactivation [16,38], or estimated a constant rate of production of virus, rather than the frequency of events [23]. Rong *et al* used a similar modeling approach to understand viral ‘blips’ during ART, and estimated that these were infrequent [39]. Pennings *et al* estimated the frequency of successful latent cell reactivation under ART as five times per day, based on the rate of development of drug resistance under ART and viral mutation rate [19], and more recently Hill *et al* used this frequency to model the affects of LRA [18]. Our estimate of the frequency of successful reactivation from latency (once every 5–8 days) is based upon analysis of the distribution in time-to-detection in the patient population, and is substantially slower than these previous estimates. The relatively slow frequency of recrudescence has important implications for understanding how to prolong anti-retroviral-free viral remission. In addition, careful consideration of the dynamics of viral recrudescence is critical to designing successful future eradication studies. Our work suggests that rather than using indirect approaches to estimate reductions in the reservoir and predict delays in time-to-recrudescence, we should measure this directly.

Previous studies of the number of latently infected cells under therapy have estimated that there are between 1 million and 60 million latently infected cells in the body, and the half-life of the latent reservoir is around 44 months [11–13]. One question that arises from this is whether reactivation from latency plays a significant role in the observed rate of decay of the latent reservoir, and whether periodic reactivation may lead to the reservoir ‘running dry’ [18,39,40]. If the current estimates of the number of latently infected cells and their decay rate are correct, this means the reservoir ‘loses’ on average at least 500 cells per day. Since we observe a successful reactivation from latency and reseeding of the viral reservoir only every 6 days, this suggests that reactivation would play a minimal role in the decay of the latent reservoir (unless the reservoir is much smaller than previously estimated). Previous modelling has assumed that there may be many ‘abortive’ reactivation events for every successful reactivation leading to recrudescence [18]. This might occur, for example, if very early events in viral reactivation are controlled by the host immune response. However even considering this possibility, it seems unlikely that reactivation from latency is a major factor contributing to the observed half-life of the latent reservoir. Finally, if we assume that previous estimates of the reservoir size are correct, we can also estimate the average probability of an individual latently infected cell successfully initiating viral recrudescence on a given day, and the average time until an individual cell is likely to achieve this. Assuming a conservative reservoir size of one million latently infected cells per patient and the fact that on average a patient has only one successful reactivation every 6 days, we can calculate that an individual latent cell has only a 1.7 x 10^−7^ probability of initiating viral recrudescence each day. Thus, the average time for an individual latent cell to initiate infection (assuming they all have the same probability of this) is around 16,500 years (ie: most latent cells will not successfully initiate an infection within the lifetime of the host). Although this per-cell probability of reactivation appears very low, it is perhaps worth considering how the reservoir is generally measured experimentally. The estimate of one million cells comes from the frequency of cells able to initiate viral growth in an *in vitro* viral outgrowth assay [12]. This assay involves stimulation of cells with PHA, and thus aims to estimate the number of ‘reactivatable’ latent cells with this strong and generalized stimulus. Reactivation *in vivo* may rely on antigenic stimulus, or random weak reactivation events, which activate a much smaller proportion of latent cells at any one time. Thus, it is not surprising that a much smaller number of cells is estimated to reactivate *in vivo*, than can be stimulated *in vitro*. Nonetheless, the *in vitro* quantitative viral outgrowth assay likely gives us a valuable measure of the size of the reservoir, as long as we recognize that only a fraction of these will actually reactivate in a given time. Given this potential for very prolonged quiescence of latent cells, it is not surprising that reactivation can be observed after prolonged periods of remission, as has been observed after transplantation and in the case of the Mississippi baby [29,30]. In our analysis we estimated a frequency of initiation of viral rebound for the different cohorts as if all patients in a cohort had the same frequency. However, recent studies have shown that reservoir size may vary substantially between patients and appears correlated with time to recrudescence after ART-interruption [20–22], and it is highly likely that our patients also differed in reservoir size and rebound rate. To investigate this, we looked at whether time-to-detection was correlated for individual patients undergoing successive ART-interruptions in the SSITT trial (cohort 4). We found that time-to-detection was indeed correlated over multiple interruptions in individual patients (Kendal’s concordance W = 0.47, *p* = 0.013). Thus, it seems likely that the frequency of reactivation we estimate for the cohorts represents the average frequency in the cohort, and there will be a distribution amongst individuals. In addition, we model reactivation as if it were the only mechanism affecting time to detection, and disregard the effects of viral growth because it is not correlated with time-to-detection. It is clear that differences in growth rates will inevitably affect time-to-detection, as slower growing virus will be seen later. However, unless the distribution of delays due to growth is large compared to the delays due to time-to-initiation, we would not expect growth to correlate well with time-to-detection (as we have recently illustrated in the context of malaria infection [41]).

There are clear limitations of our analysis of time-to-detection after ART-interruption, including the use of diverse cohorts, capturing patients at different times of infection, or after different interventions (including the use of an LRA)(summarised in Table 1). Our analysis was limited to ART-interruption studies with regular sampling after interruption, as this is required to capture the time-to-detection. Despite these obvious differences in the cohorts, we find very similar estimates of the average frequency of reactivation. These estimates were confirmed by a completely different approach, analyzing the ratios of ‘reactivation founder’ virus after ART-interruption. Our analysis suggests that much larger and more frequently sampled cohorts may be required to demonstrate differences in time-to-recrudescence amongst patients treated at different stages of infection or for different times (consistent with the predictions of the power analysis). One apparent paradox of any estimate of frequency of reactivation is the observation of persistent low viral loads in patients on ART [42,43]. If infectious virus were continuously present, then there is no real concept of delay-to-reactivation (and this argument applies equally to estimates of five reactivation events per day, or one every 6 days). However, the presence of reactivation founder virus suggests that viral growth is initiated by discreet reactivation events, rather than a constant ‘dribbling’ of virus from the latent reservoir [25]. Therefore it seems likely that the low levels of circulating virus detected under ART do not provide an immediate source of virus for reactivation.

The development of therapies to purge the latent reservoir of HIV and produce prolonged antiretroviral-free HIV remission is a major priority. Understanding the frequency of recrudescence from latency is a crucial parameter in predicting the impact of interventions. Establishing the ‘normal’ rate of recrudescence from latency in HIV also allows us to assess the appropriateness of animal models and interventions, which can be judged on their ability to alter this parameter. A variety of approaches have been proposed to assess the effectiveness of latency reversing drugs. However, ultimately the test of LRA efficacy is the length of remission after ART-interruption. Future studies should determine the best predictors of time-to-recrudescence, so that these measures may be used as proxies to assess the efficacy of HIV eradication interventions.

## Materials and Methods

### Ethics statement

This manuscript involves the analysis of previously published data from original human and animal studies published elsewhere (summarized in Table 1). Details of the ethical approval for the original studies may be found in the original publications.

### Modelling frequency of initiation of viral replication

To study the dynamics of viral recrudescence, we assumed that the initiation of viral replication after ART interruption is a random event, occurring at a given frequency. Thus, the time-to-initiation will be exponentially distributed, and the proportion of patients without reactivation (*P*) will follow the equation:

$$P=P_{0}e^{-k(t-t_{0})}$$ (2)

Where *P*_*0*_ is the initial number of patients, *k* is the frequency of reactivation (ie: reactivation occurs once every 1/*k* days), and *t*_*0*_ is the minimal time to detection (as a result of ART-washout and the time taken for virus to grow from the initial level of viral infection to the level of detection (summarized in Fig 1). The equation was fitted to the data using the least squares method.

In order to compare rates of reactivation between studies, we allowed the initial delay to detection to be an independent parameter for each study (since both the ART drugs and threshold of detection varied between groups), and estimated the optimal frequency of recrudescence (*1/k*) across all groups. To investigate whether the frequency of recrudescence (*1/k*) was significantly different between groups we used an F-test.

### HIV growth rate during reactivation

In order to estimate whether differences in viral growth could account for the observed delays to detection, we estimated viral growth rates from the viral load data, assuming exponential growth of the virus. We then investigated whether growth rate was correlated with time-to-detection, as would be expected if delayed detection occurred due to slower viral growth. In the first cohort of 9 patients sampled frequently, we used linear regression to estimate the slope of log-transformed viral load with time, using 2–5 sequential viral load measurements. In the second cohort of 59 patients sampled weekly, we estimated viral growth rate using a two-point estimate of the growth between the first and second positive viral load samples. Note that this may tend to underestimate viral growth if it growth slows as viral load increases. Moreover, there will be a tendency for patients detected with a lower viral load to have a faster growth rate (because growth is measured at an earlier (and thus faster) stage of infection). The observation of lower viral loads in patients detected in the first week (Fig 3E) is likely an artefact of the pharmacokinetic delays before drug was fully eliminated and viral growth was possible in the first days after interruption.

An additional assumption in our analysis is that the viral growth rate in plasma at the time of detection is reflective of (or at least proportional to) viral growth early after viral reactivation.

### Estimating the statistical power of interruption and time-to-detection studies

In order to detect statistically significant differences of hazard ratios (*HR*) by Log-Rank Test with the level of significance α and power 1-β we need to have sufficient number of patients in each arm of the experiment. For estimation of this number we first need to estimate the number of events (recrudescence of virus) (*m*) and for this purpose used a formula, which assumes the equal number of patients in each arm [44];

$$m=4{\left(z_{\alpha /2}+z_{\beta }\right)}^{2}/\theta$$ (3)

where θ = Ln(*HR*).

However, if the rate of detection is not high enough to observe all patients in given time window of follow up, then the number of events will be lower than the total number of patients. Thus we need to correct the value of *m* by the fraction of patients with detectable virus at the end of the study. Assuming the exponential time to detection with the rate of detection estimated in our study (*k*) we can write the formula that relates the reduction in reactivation rate and the number of patients in one arm of the study.

$$n=\frac{4{\left(z_{\alpha /2}+z_{\beta }\right)}^{2}}{\text{ln}{\left(1-p/100\right)}^{2}(1-e^{-kt})(1-e^{-\frac{p}{100}kt})}$$ (4)

where *p* is the percent reduction in reactivation rate, *t* is the time window of analysis.

### Modeling the ratios of reactivation founder virus

Sequence data on viral quasispecies after ART-interruption from the Joos study were obtained from Genbank (Genbank accession numbers listed in the original publication [25]). The data were analysed using a ‘highlighter plot’ (www.hiv.lanl.gov) to identify the relationships between different viral species within a given patient (see S3 File). Six patients were identified in whom we could distinguish and count the frequency of founder viruses early after ART-interruption, and this data was used to find the ratio of the number of copies of each observed founder virus in a patient to the next largest founder.

To estimate the frequency of reactivation from the ratio of founder viruses, we assumed an exponential time-to-initiation of viral growth, and exponential growth of virus during the initial phase of infection. We can then write down a formula for the expected ratios (*R*) between the sizes of subsequent founders:

$$R=\frac{V_{0}e^{gt_{1}}}{V_{0}e^{gt_{2}}}=e^{g\Delta }$$ (5)

Where *g* is the growth rate of virus (= 0.4 day^-1^), Δ = *t*_1_ ‒*t*_2_ is the delay between successive initiation events at times *t*_1_ and *t*_2_, and *V*_0_ is the initial concentration of virus. The distribution of delays between the initiation of growth of different founders (and thus their ratios) will be determined by the frequency of initiation of viral growth after ART-interruption. We assume that that Δ has an exponential distribution with parameter λ and can then derive a formula for the probability density function (PDF) of the expected ratios (h(*y*)) using the formula for distribution function of a random variable.

$$h(y)=\;\frac{f_{\text{exp}}(\lambda ,\text{ ln}(y)/g)}{yg},$$ (6)

Where f_exp_(λ, *x*) is the probability density function (PDF) of the exponential distribution. The cumulative distribution function (CDF) of the ratios, H(*y*), can be defined by:

$$H(y)=\;F_{\text{exp}}(\lambda ,\text{ ln}(y)/g)$$ (7)

Where F_trexp_(λ, *x*) is the CDF of the exponential distribution.

By using maximum likelihood estimation to fit the observed ratios between the number of copies of founders to h(*y*) we are able to estimate the rate of successful reactivation λ.

We note that this analysis implicitly assumes that different founders grow at the same rate. It is also possible that individual founder viruses grow at different rates. However, as long as the growth rate is independent of the reactivation time, this should not significantly affect the expected distribution of founder ratios.

## Supporting Information

S1 File Modelling the impact of stochastic delays. (PDF)

S2 File Modelling the effects of different initial levels of virus. (PDF)

S3 File Analysis of founder ratios. (PDF)
