# Correction: HIV Reactivation from Latency after Treatment Interruption Occurs on Average Every 5-8 Days—Implications for HIV Remission

**DOI:** 10.1371/journal.ppat.1005745

**Published:** 2016-08-25

**Authors:** Mykola Pinkevych, Deborah Cromer, Martin Tolstrup, Andrew J. Grimm, David A. Cooper, Sharon R. Lewin, Ole S. Søgaard, Thomas A. Rasmussen, Stephen J. Kent, Anthony D. Kelleher, Miles P. Davenport

There are errors in the data analysis of several patients in cohort 4, interruption 1, that affect the reactivation rate for cohort 4 reported in the Results section and Fig 4.

**Fig 4:**
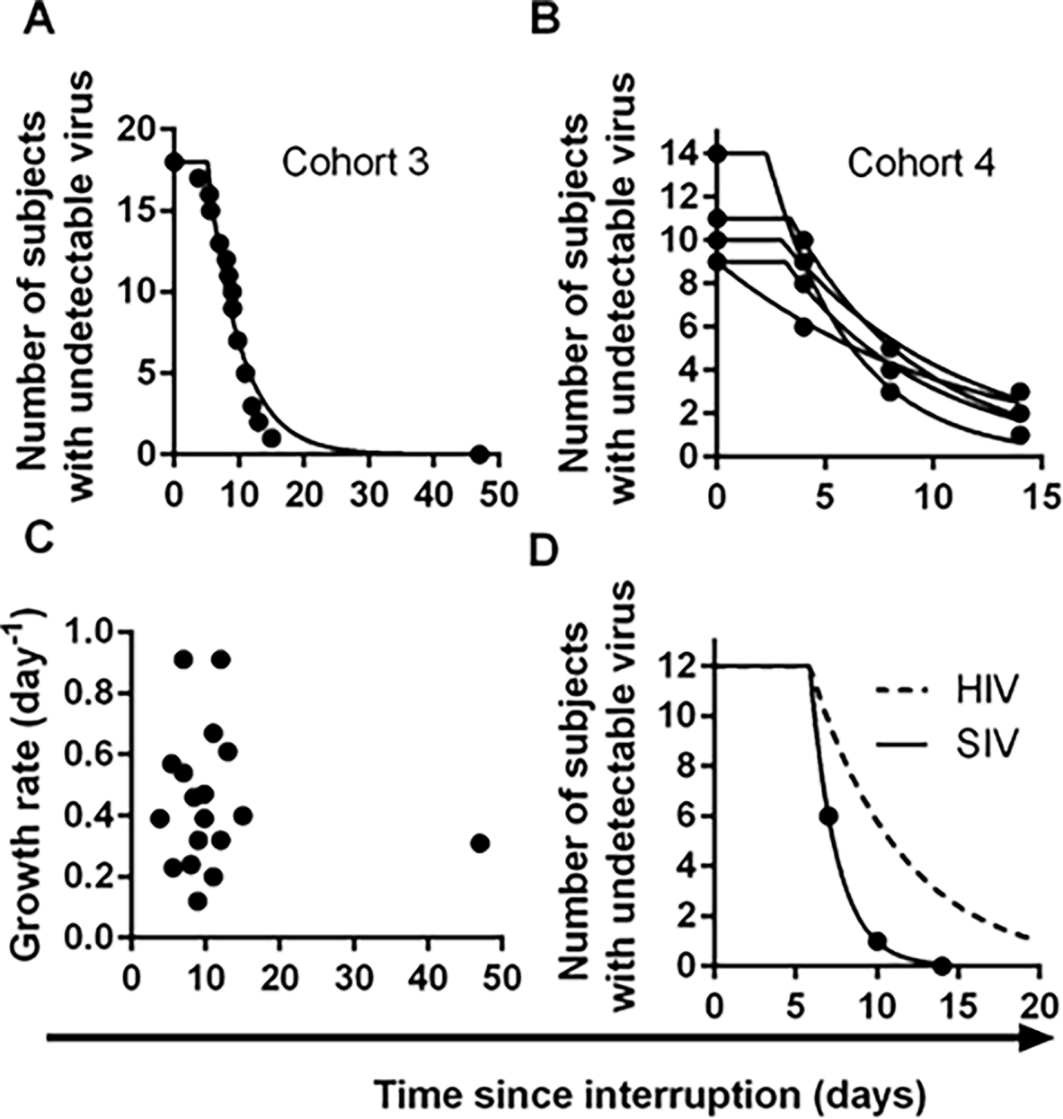
Time-to-detection of virus in cohorts 3 and 4. (A) time-to-detection in cohort 3, of 18 patients undergoing interruption (reference [16]). The best-fit frequency of reactivation is once every 5.1 days. (B) time-to-detection in cohort 4, of 14 patients undergoing five interruptions, and monitored at days 4, 8, and 14 (reference [17]). The best-fit frequency over all interruptions is once every 6.3 days. (C) Time to recrudescence is not correlated with growth rate in cohort 3. (D) Higher reactivation rates in SIV than HIV. The estimated frequency of initiation of viral replication in SIV infected macaques treated with ART between 7 and 14 days post-infection (from reference [26]) is shown as solid line, and was found to be once every 1.7 days. The best-fit frequency of reactivation across the four HIV cohorts (a reactivation event every 6 days) is shown as a dashed line.

The data for cohort 4 was extracted from Fig 1A of reference 17. This involves 14 individuals undergoing 5 sequential treatment interruptions. Data for time-to-detection after treatment interruption for cohort 4 of our paper (displayed in Fig 4B) was extracted directly from this table. Patients who had detectable virus at the time of interruption (for any given interruption) were excluded from analysis in that interruption.

Unfortunately in interruption 1, patients were accidentally excluded if they had detectable HIV at the time of interruption for any of the five interruptions (instead of being excluded only if they had detectable HIV in the first interruption). Thus, instead of 14 patients that should have recorded a time-to-detection in interruption 1, 6 patients were wrongly excluded (patients 109, 112, 116, 125, 127, 129) and only 8 patients were included in this analysis for interruption 1. In interruptions 2 to 5, patients were correctly excluded.

The inclusion of these additional 6 patients for interruption 1 alters the best estimate of frequency of reactivation for cohort four from once every 7.2 days to once every 6.3 days. We note that inclusion of interruption 1 for these 6 patients does not affect the average rate of reactivation over all patients in the four cohorts (once every 6.0 days), as this only affects one reading in 6 out of 100 patients analyzed.

The number 7.2 should be changed to 6.3 days in the fourth sentence of the second paragraph and last sentence of the last paragraph of the subsection "HIV reactivation in other cohorts", as well as in the Fig 4B legend. Please find the corrected Fig 4 here. This error does not affect the conclusions of the paper.
