# Supplementary material for: HIV Reactivation from Latency after Treatment Interruption Occurs on Average Every 5-8 Days—Implications for HIV Remission
Source: PLoS Pathog. 2015 Jul 2;11(7):e1005000. doi: 10.1371/journal.ppat.1005000 (PMC4489624; doi:10.1371/journal.ppat.1005000)
Supplement: S1 File — (PDF) [file ppat.1005000.s001.pdf]

S 1. Multiple recrudescence of virus shortens the stochastic delay before the exponential growth Phase.

Early stochastic events may play a role in delaying the time-to-detection of virus after an initial reactivation event. This may occur because, when there are only small numbers of infected cells, it is possible for the number of infected cells to expand, contract, or occasionally continue at low levels for some time before expanding (leading to a delay before exponential growth commences). Here we analyse data from a previous study of stochastic delay, and show that the delay from the first rebound of virus to the exponential growth phase is generally small, and also that it decreases with increasing frequency of viral reactivation.

In order to illustrate the effects of rebound frequency on stochastic delay, we will consider only the first two consecutive rebounds after ART-interruption. Let X_i be the random variable that defines the delay due to stochastic processes of the i -th virus reactivation, and the distribution of X_i is defined by CDF $F_i(x)$, i=1,2. Since we detect the virus with the shortest stochastic delay X_i (assuming the same growth rate, after the stochastic part), we need to find the distribution function G(Y) of the random variable $Y=\min(X_1, X_2)$. Using the definition of the distribution function and assuming that we can write the following derivations given X_i are independent, i=1,2:

$$\begin{aligned} G(y) &= P(Y < y) = 1 - P(Y > y) = 1 - P(X_1 > y, X_2 > y) = \\ &= 1 - \prod_{i=1}^{2} P(X_1 > y) = 1 - \prod_{i=1}^{2} (1 - F_i(y)). \end{aligned} \qquad \text{(S1.1)}$$

We first sought to approximate the distribution of delays due to stochastic events expected from a single reactivation event, using the distribution derived by Pearson *et al* (18) and approximating this with a lognormal distribution with parameters μ= 0.513 and σ= 0.307 which is correspond to the mean delay of 1.75 days and standard deviation of 0.55 days (extracted from the Figure 11 in reference 18).

Thus X_1 has a lognormal distribution. However, X_2 incorporates the delay until the second rebound of virus, so $X_2 = S_1 + E_1$, where S_1 is distributed lognormally and E_1 has exponential distribution with the parameter λ corresponding to daily rate of rebound. The CDF of X_2 will be the convolution of CDF of exponential distribution and PDF of lognormal distribution.

$$F_2(t) = \int_0^t F_{\exp}(\lambda, t - x) f_{\ln}(\mu, \sigma, x) dx, \;. \qquad \text{(S1.2)}$$

Where $F_{exp}(\lambda, i, t)$ is CDF of an exponential distribution with the rate parameter λ and $f_{ln}(\mu, \sigma, x)$ is the PDF of a lognormal distribution with parameters μ and σ.

Variables X_1 and X_2 are independent so we can apply formula (S1.1) to find the distribution of minimal delay (Figure S1.1).

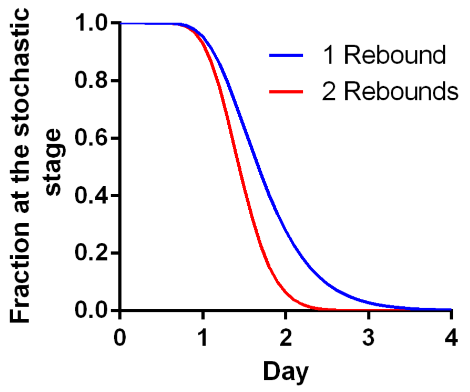

Figure S1.1. The fraction of patients with a given duration of the stochastic phase. If we consider only the delay from the first viral reactivation, then we would expect around 1/3 of reactivations to have a delay of 3 days or greater (blue line). However, if we have a high frequency of reactivation, then it is likely that the second reactivation has a shorter stochastic delay than the first, and actually starts exponential growth first. At a rebound rate $k = 5$ day^{-1}, the probability of a stochastic delay of >2 days becomes much smaller, and the expected duration of the stochastic phase shortens with an increasing number of rebounds considered.
