# Supplementary material for: HIV Reactivation from Latency after Treatment Interruption Occurs on Average Every 5-8 Days—Implications for HIV Remission
Source: PLoS Pathog. 2015 Jul 2;11(7):e1005000. doi: 10.1371/journal.ppat.1005000 (PMC4489624; doi:10.1371/journal.ppat.1005000)
Supplement: S2 File — (PDF) [file ppat.1005000.s002.pdf]

S2. Impact of changing initial 'seed' level of of virus on the delay to detection.

Let us assume that growth of virus on initial growth phase can be described by exponential growth function:

$$V(t) = V_0 e^{gt} \qquad \text{(S0.1)}$$

Where V is the concentration of virus, V_0 is the initial seed of virus, g –growth rate. We can rewrite the formula (S0.1) with respect to V_0.

$$\frac{V_0}{T} = \frac{1}{e^{gt}} \qquad \text{(S0.2)}$$

Where is T is the detection threshold. Assuming that V_0^1 produces delay t_1 and V_0^2 produces delay t_2 we can write the relation between change in V_0 and t.

$$\frac{V_0^1}{V_0^2} = e^{g(t_2 - t_1)} \qquad \text{(S0.3)}$$

Thus, we can see that the increase in time to detection by $x = t_1 – t_2$ days requires an exponential decrease of V_0 (Figure S2.1).

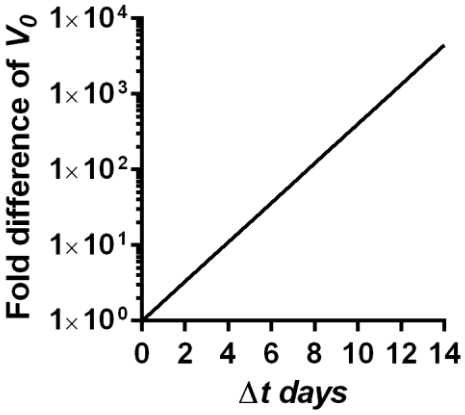

Figure S2.1. Relationship between fold difference in the initial seed of virus V_0 and increase in corresponding time to detection Δt . Even assuming conservative initial growth rate g=0.6,we obtain 4.4×10^3 fold difference in V_0 that is necessary to produce the delay between detection of two weeks.
