# Supplementary material for: HIV Reactivation from Latency after Treatment Interruption Occurs on Average Every 5-8 Days—Implications for HIV Remission
Source: PLoS Pathog. 2015 Jul 2;11(7):e1005000. doi: 10.1371/journal.ppat.1005000 (PMC4489624; doi:10.1371/journal.ppat.1005000)
Supplement: S3 File — (PDF) [file ppat.1005000.s003.pdf]

S3. Analysis of founder ratios.

Joos et al [1] analysed viral rebound in 20 patients undergoing ART-interruption. Of these, nine patients had sequencing data early after interruption, and 11 patients only had sequence data after prolonged interruption. Of the nine patients with early data, three were excluded because their pre-ART viral population was relatively homogeneous, and thus there was a high risk that two reactivated latent cells may contain the same viral sequence and be misclassified as a single founder. The sequences from the remaining six patients were aligned using a highlighter plot to identify individual 'founder' viruses. As seen in studies of founder virus in primary infection, some diversification had arisen between viral reactivation and the sampling of virus. Thus, founder progeny were not always identical at all nucleotides, but may have differed by one or a few nucleotides from the (presumed) dominant original founder sequence.

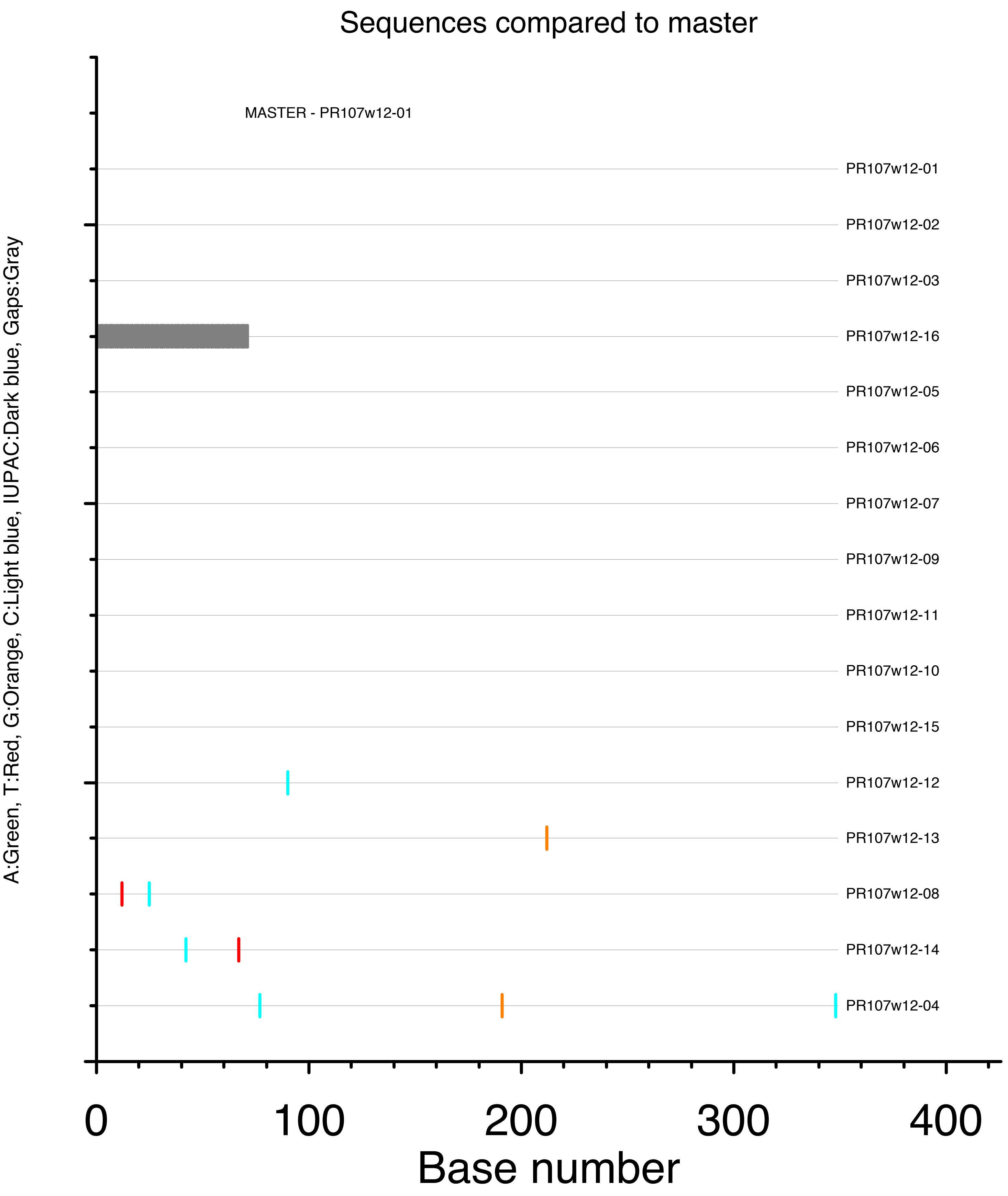
Sequences compared to master
MASTER - PR107w12-01
PR107w12-01
PR107w12-02
PR107w12-03
PR107w12-16
PR107w12-05
PR107w12-06
PR107w12-07
PR107w12-09
PR107w12-11
PR107w12-10
PR107w12-15
PR107w12-12
PR107w12-13
PR107w12-08
PR107w12-14
PR107w12-04
A:Green, T:Red, G:Orange, C:Light blue, IUPAC:Dark blue, Gaps:Gray
0
100
200
300
400
Base number

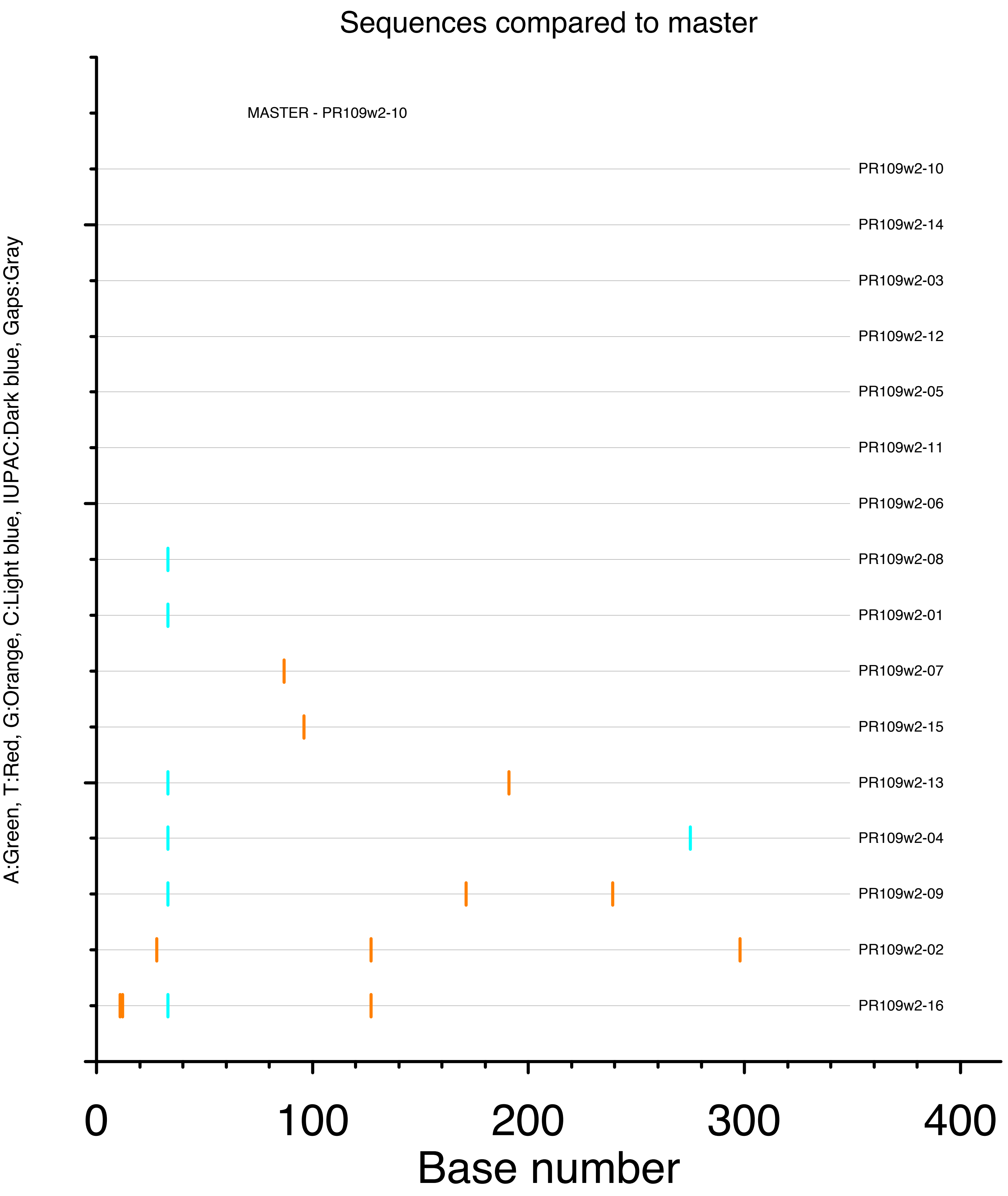
Sequences compared to master
MASTER - PR109w2-10
PR109w2-10
PR109w2-14
PR109w2-03
PR109w2-12
PR109w2-05
PR109w2-11
PR109w2-06
PR109w2-08
PR109w2-01
PR109w2-07
PR109w2-15
PR109w2-13
PR109w2-04
PR109w2-09
PR109w2-02
PR109w2-16
A:Green, T:Red, G:Orange, C:Light blue, IUPAC:Dark blue, Gaps:Gray
0
100
200
300
400
Base number

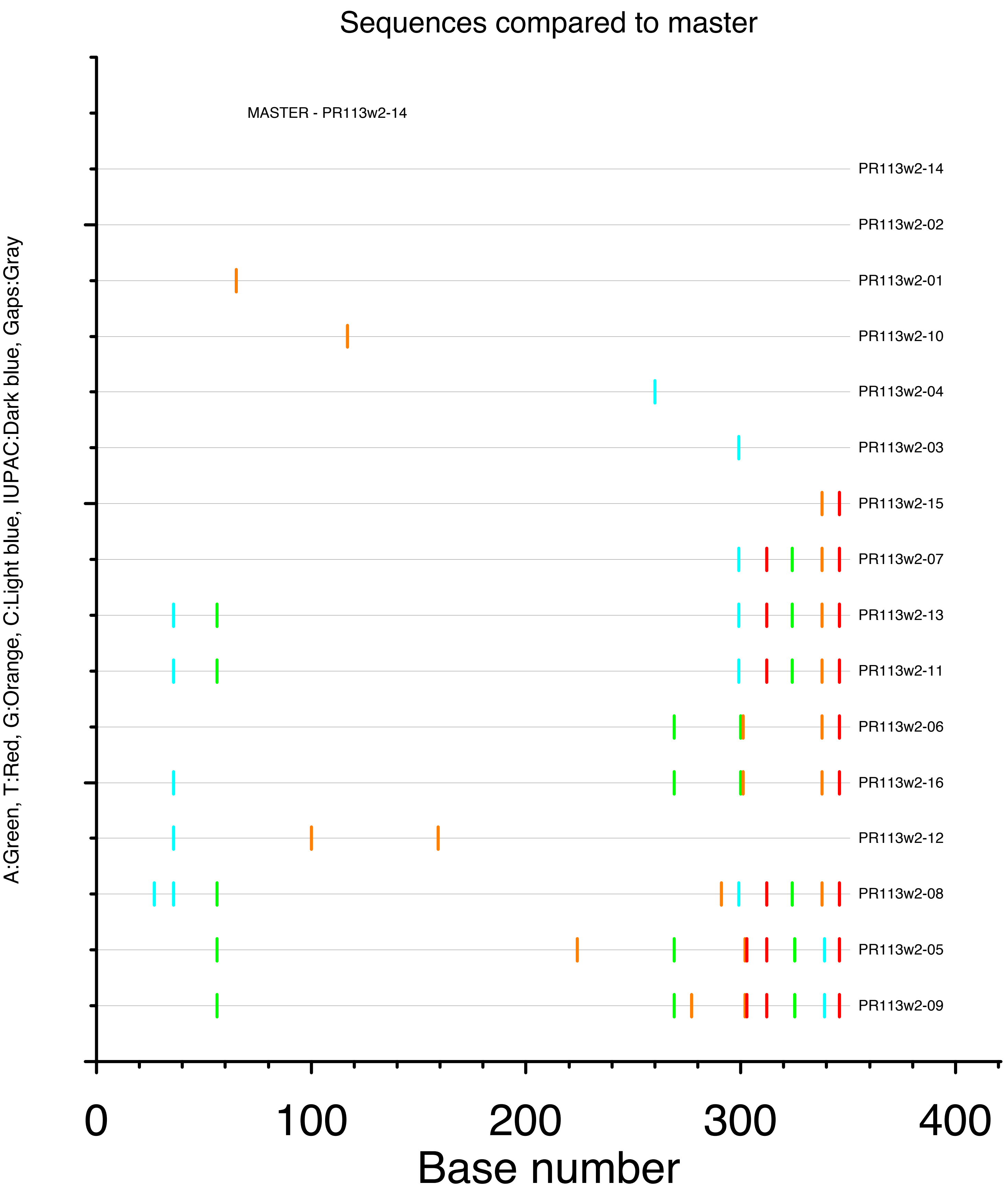
Sequences compared to master
MASTER - PR113w2-14
PR113w2-14
PR113w2-02
PR113w2-01
PR113w2-10
PR113w2-04
PR113w2-03
PR113w2-15
PR113w2-07
PR113w2-13
PR113w2-11
PR113w2-06
PR113w2-16
PR113w2-12
PR113w2-08
PR113w2-05
PR113w2-09
A:Green, T:Red, G:Orange, C:Light blue, IUPAC:Dark blue, Gaps:Gray
0
100
200
300
400
Base number

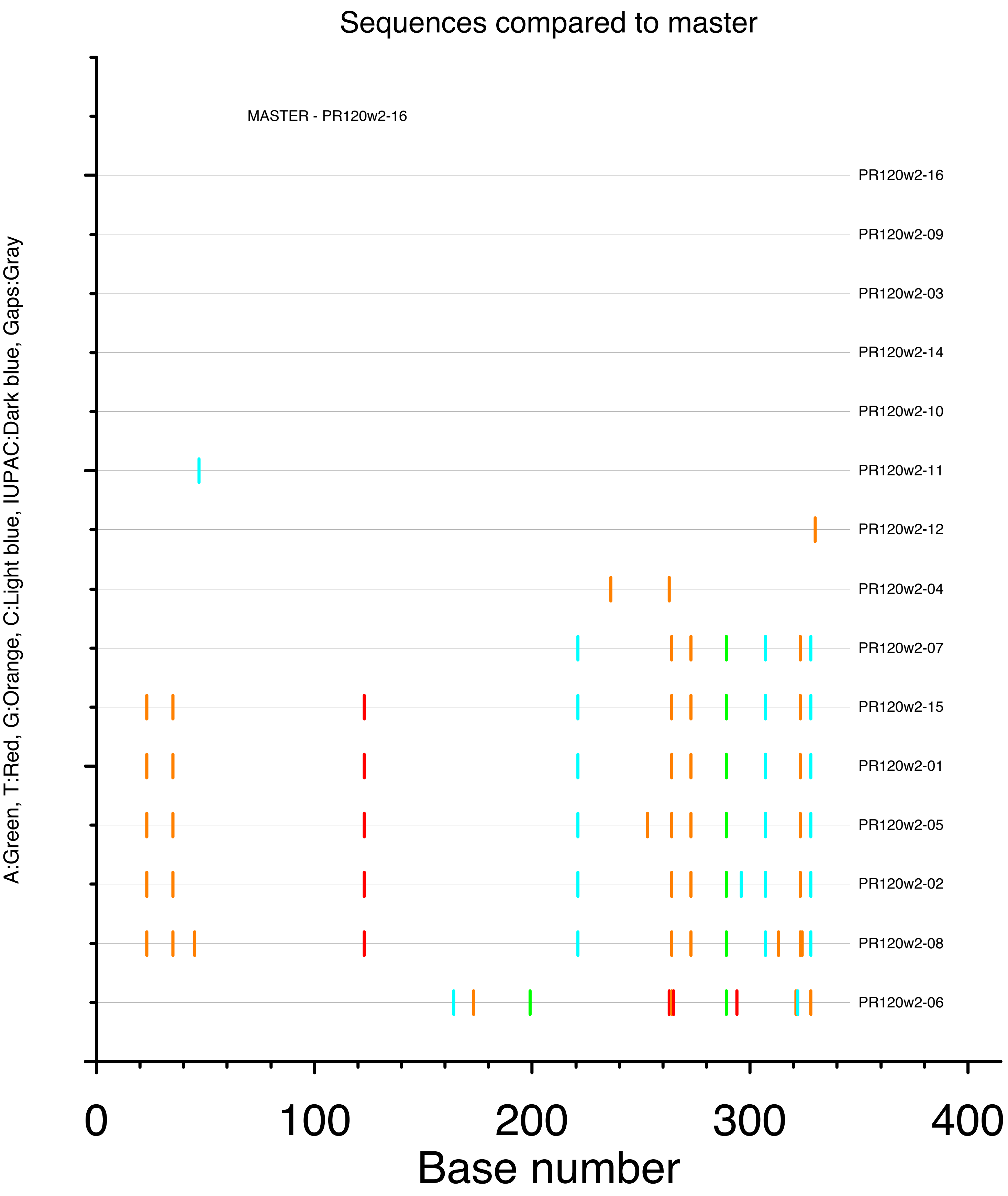
Sequences compared to master
MASTER - PR120w2-16
PR120w2-16
PR120w2-09
PR120w2-03
PR120w2-14
PR120w2-10
PR120w2-11
PR120w2-12
PR120w2-04
PR120w2-07
PR120w2-15
PR120w2-01
PR120w2-05
PR120w2-02
PR120w2-08
PR120w2-06
A:Green, T:Red, G:Orange, C:Light blue, IUPAC:Dark blue, Gaps:Gray
0
100
200
300
400
Base number

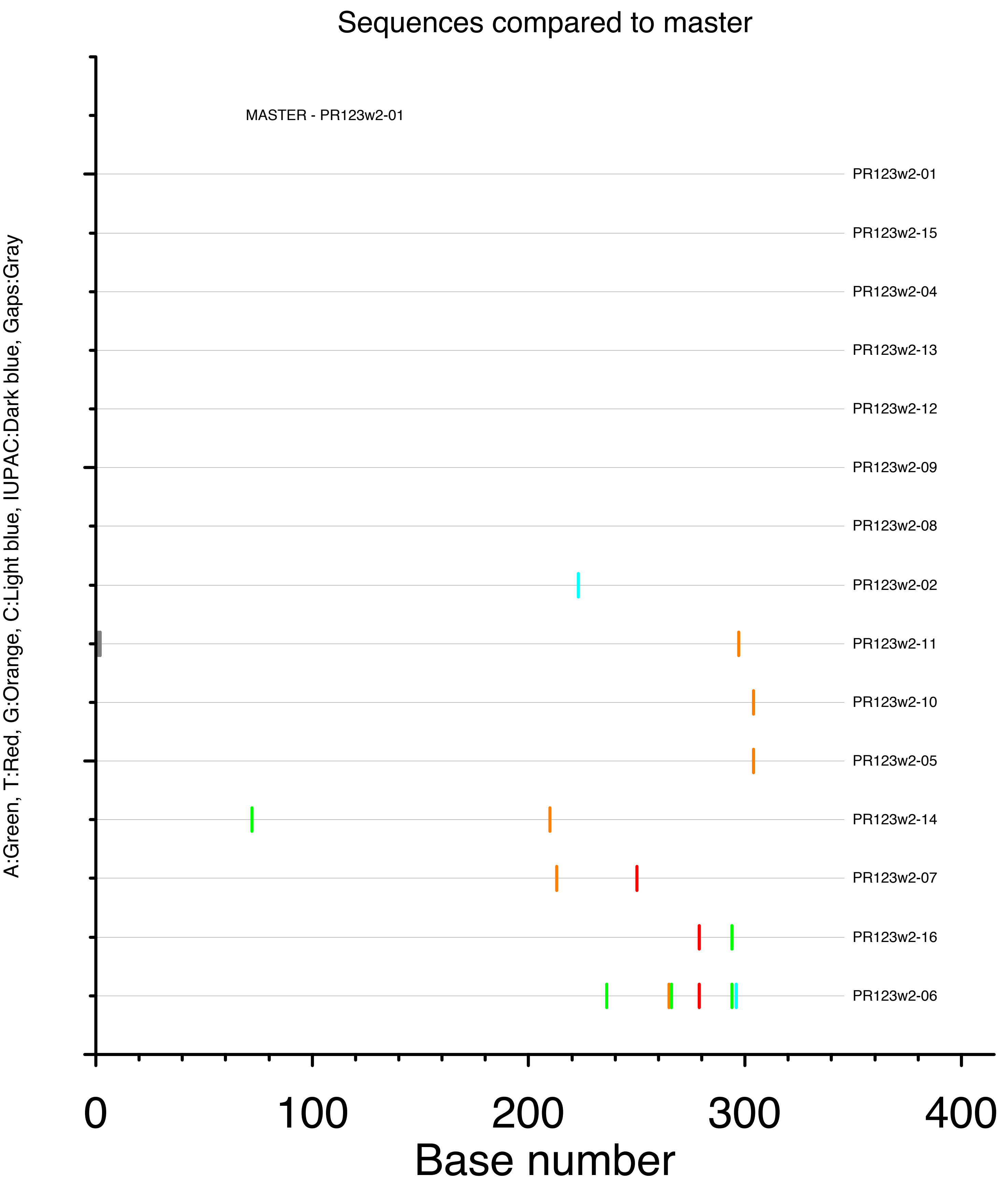
Sequences compared to master
MASTER - PR123w2-01
PR123w2-01
PR123w2-15
PR123w2-04
PR123w2-13
PR123w2-12
PR123w2-09
PR123w2-08
PR123w2-02
PR123w2-11
PR123w2-10
PR123w2-05
PR123w2-14
PR123w2-07
PR123w2-16
PR123w2-06
A:Green, T:Red, G:Orange, C:Light blue, IUPAC:Dark blue, Gaps:Gray
0
100
200
300
400
Base number

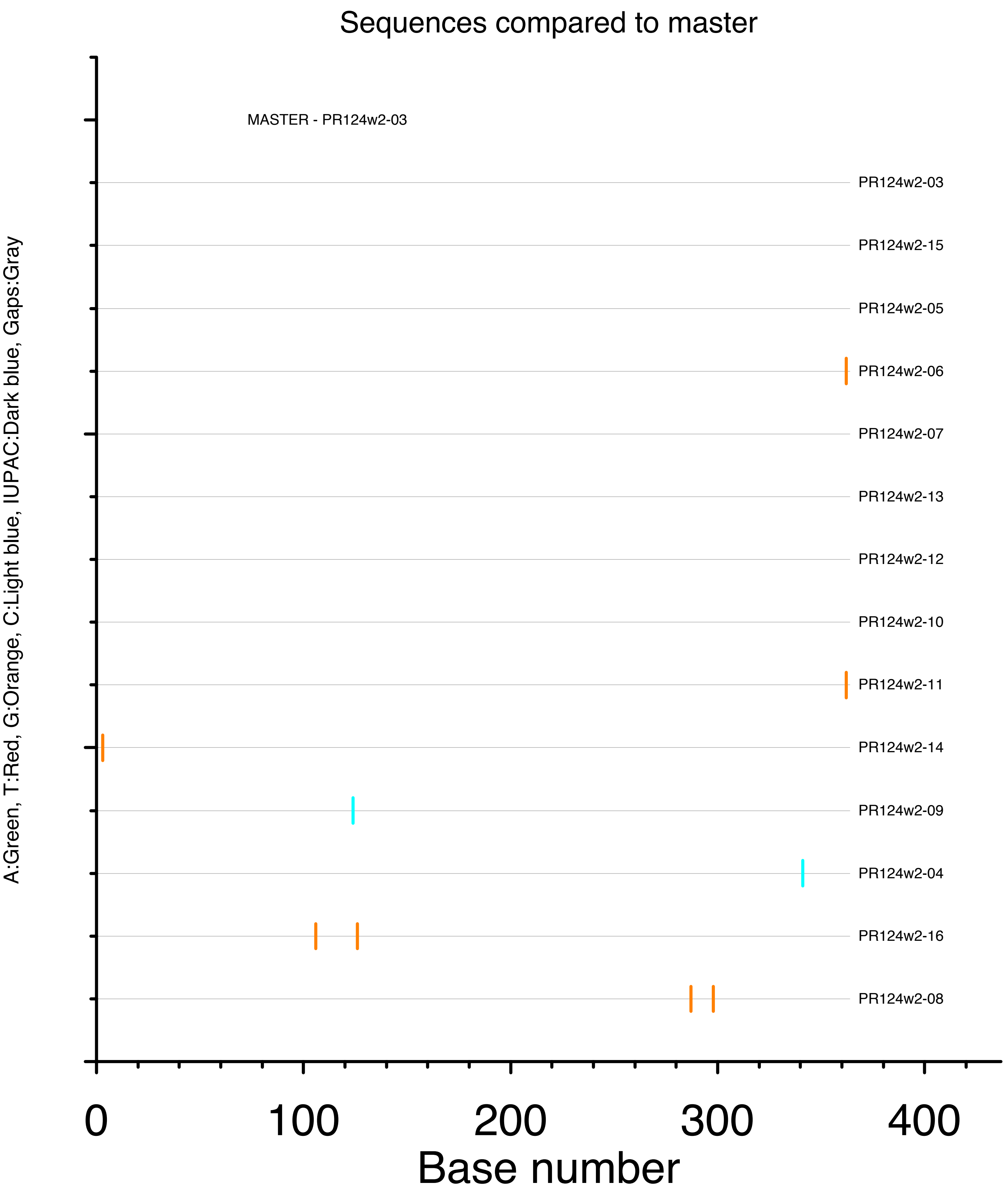
Sequences compared to master
MASTER - PR124w2-03
PR124w2-03
PR124w2-15
PR124w2-05
PR124w2-06
PR124w2-07
PR124w2-13
PR124w2-12
PR124w2-10
PR124w2-11
PR124w2-14
PR124w2-09
PR124w2-04
PR124w2-16
PR124w2-08
A:Green, T:Red, G:Orange, C:Light blue, IUPAC:Dark blue, Gaps:Gray
0
100
200
300
400
Base number

1. Joos B, Fischer M, Kuster H, Pillai SK, Wong JK, et al. (2008) Proc Natl Acad Sci U S A 105: 16725-16730.
